# Loss of heterozygosity at D8S262: an early genetic event of hepatocarcinogenesis

**DOI:** 10.1186/s13000-015-0308-y

**Published:** 2015-06-16

**Authors:** Qiao Zhu, Li Gong, Xiaoyan Liu, Jun Wang, Pin Ren, Wendong Zhang, Li Yao, Xiujuan Han, Shaojun Zhu, Miao Lan, Yanhong Li, Wei Zhang

**Affiliations:** The Helmholtz Sino-German Laboratory for Cancer Research, Department of Pathology, Tangdu Hospital, the Fourth Military Medical University, Xi’an, 710038 People’s Republic of China; Department of Rehabilitation Medicine, Tangdu Hospital, the Fourth Military Medical University, Xi’an, 710038 People’s Republic of China; Department of Gynaecology and Obstetrics, Tangdu Hospital, the Fourth Military Medical University, Xi’an, 710038 People’s Republic of China

**Keywords:** Dysplastic nodules, hepatocellular carcinoma, loss of heterozygosity, D8S262, CSMD1

## Abstract

**Background:**

Hepatocellular carcinoma (HCC) is a multi-factor, multi-step, multi-gene and complicated process resulting from the accumulation of sequential genetic and epigenetic alterations. An important change among them is from precancerous lesions to HCC. However, only few studies have been reported about the sequential genetic changes during hepatocarcinogenesis.

**Methods:**

We observed firstly molecular karyotypes of 10 matched HCC using Affymetrix single-nucleotide polymorphism (SNP) 6.0 arrays, and found chromosomal fragments with high incidence (more than 70 %) of loss of heterozygosity (LOH). Then, we selected 28 microsatellite markers at some gene spanning these chromosomal fragments, and examined the frequency of LOH of 128 matched HCC and 43 matched precancerous lesions-dysplastic nodules (DN) by a PCR-based analysis. Finally, we investigated the expression of proteins encoded by these genes in HCC, DN and the surrounding hepatic tissues.

**Results:**

The result of Affymetrix SNP6.0 arrays demonstrated that more than 70 % (7/10) cases had chromosomal fragment deletion on 4q13.3-35.1, 8p23.2-21.2, 16q11.2-24.3, and 17p13.3-12. Among 28 microsatellite markers selected, LOH frequencies at D8S262 for DN and HCC were found to be the highest, 51.2 % and 72.7 %, respectively. Immunohistochemically, the positive rate of its adjacent gene CSMD1 in HCC, DN, and the surrounding hepatic tissues were 27.3 % (35/128), 75 % (33/44), and 82 % (105/128), respectively.

**Conclusions:**

LOH at D8S262 may be associated with an early genetic event of hepatocarcinogenesis, and a predictor for the monitor and prevention of HCC.

**Virtual Slides:**

The virtual slides for this article can be found here: http://www.diagnosticpathology.diagnomx.eu/vs/1557074981159099.

**Electronic supplementary material:**

The online version of this article (doi:10.1186/s13000-015-0308-y) contains supplementary material, which is available to authorized users.

## Background

Hepatocellular carcinoma (HCC) is the fifth most common cancer and the third most common cause of cancer-related deaths worldwide [1]. Up to 80 % of HCCs develop against a background of liver cirrhosis related to hepatitis B and C virus infections. Moreover, precancerous lesions of HCC, such as dysplastic foci (DF), including small cell change (SCC) and large cell change (LCC), dysplastic nodules (DN), including low grade DN (LGDN) and high grade DN (HGDN), and nodules of altered hepatocytes (NAHs) described previously by Su et al. [2, 3] are often found in cirrhotic liver tissue. Therefore, mounting evidence suggests that SCC represents precursor lesions that are more advanced than LCC in the course of human hepatocarcinogenesis [4]. Foci of altered hepatocyte (FAH) is observed in several animal species in the early stages of hepatocarcinogenesis caused by chemicals, radiation and chronic infection with hepadnaviruses. FAH is usually composed of 10–1000 cells, and located in one or more of the hepatic lobules. It is usually more than 1 mm^2^ in cross-sectional area and compresses the surrounding parenchyma. Similar lesions have been observed fortuitously in the liver of women with long-term use of oral contraceptives and in other pathologic conditions [5–8], particularly in genetic haemochromatosis. It is classified as NAH if the whole or most of a regenerative nodule is occupied with a growth predominated FAH [3]. Our previous studies have demonstrated that part of NAH without SCC and all NAH with SCC in liver cirrhosis tissue may represent monoclonal hyperplasia. The occurrence of SCC is a late event during NAH progression, and a premalignant morphologic phenotype [9]. These observations further support the contention that NAH is a precancerous lesion of HCC. However, many professional scholars considered that the histopathological characteristics of NAH was similar to that of DN when our previous studies were reviewed by them [10], and it should be classified as DN according to their comments and WHO criteria. As the scheme of preneoplastic and neoplastic nodules during hepatocarcinogenesis described by Park [11], these lesions develop gradually into early HCC, which corresponds to *in situ* or microinvasive carcinoma, then develop into progressive HCC through the stage of “nodule-in-nodule”-type HCC. Moreover, we applied array-CGH to examine the chromosomal abnormalities of 12 monoclonal DN. The results revealed that there were some changes in DNA copy number in four chromosomal regions in one DN with SCC. Namely an increase of DNA copy number was frequently detected at 1q25.2-q21.2, 8q and 19q13.43-q13.12, while a decrease of DNA copy number was often observed at 4p, 4q and 8p. In addition, some of the chromosomal aberrations coincided with those found in HCC. However, there were no chromosomal abnormalities in another 11 DN without SCC [9]. Thus we believe that surveillance of the at-risk cirrhotic population could aid earlier detection of the disease and decrease the cancer-related mortality rate, but we are limited by the lack of sensitive biomarkers and reliable histopathological features of precancerous lesions.

Recently, with the advances in biotechnology, genome-wide analysis has provided a great deal of information for identification of candidate genes that may be involved in carcinogenesis or cancer progression. Single-nucleotide polymorphism (SNP) arrays have been used to detect genome-wide abnormalities, such as copy number changes that include loss of heterozygosity (LOH), deletions, and gene amplification events in various types of cancer, and localization of the regions of many oncogens and tumor suppressor genes (TSGs) [12–14]. Notably, the inactivation of TSGs has been shown to play an important role in hepatocarcinogenesis [15]. Allelic deletion manifested as LOH at polymorphic loci is recognized as a hallmark of TSGs, whose other allele is inactivated by point mutations, methylation or by some other mechanism [16]. The delineation of such genetic alterations that occur in precancerous lesions and/or early HCC may be important for monitoring and preventing the occurrence of HCC. Thus, we investigated molecular karyotypes of 10 matched HCC using oligonucleotide genotyping Affymetrix single-nucleotide polymorphism (SNP) 6.0 arrays, and selected the gene with high incidence of LOH to validate further by a great deal of samples, including precancerous lesions and HCC, by a PCR-based analysis.

## Methods

### Samples

Liver tissue samples from 128 cases of surgically resected HCC (male, *n* = 108; females, *n* = 20; average age = 52 years), including 39 cases of HCC without liver cirrhosis and 89 cases of HCC with liver cirrhosis, were collected between January 2007 and December 2011 from Tangdu Hospital, the Fourth Military Medical University (Xi’an, China). The study protocol was approved by the Medical Ethics Commission of the Fourth Military Medical University in Xi’an, China. Written informed consent from all participants involved in our study was obtained. All the samples resected surgically were divided into two parts, and one part was immediately stored at −80 °C, and the other was fixed in 10 % neutral formalin and embedded in paraffin. Then, serial sections were stained with haematoxylin and eosin (H/E). Each case was examined by three pathologists and diagnosed according to the morphological criteria of liver cirrhosis and HCC. HCC samples were graded according to Edmondson’s criteria. 43 DN were selected from their adjacent HCC according to the criteria described by WHO. Every HCC matched its corresponding DN. None of the patients had received any other therapies such as chemoembolization or chemotherapy before surgery.

### Laser microdissection and DNA extraction

All the samples stored at −80 °C were firstly made into frozen section, and HCC and liver cirrhosis tissues with typical histopathological characteristics were observed and selected, respectively. Then eight serial 10-μm tissue sections were prepared and placed on UV-absorbing membrane for laser microdissection by LMD6000 (Leica Microsystems Ltd, Wetzlar, Germany). After HE-staining, the slides were mounted on microstat, and the selected HCC lesion and their adjacent DN were dissected by a UV laser in mode of motorized optical beam scanning, respectively. The dissectate (with the attached specimen) dropped by its gravity into the cap of a 0.5-mL microcentrifuge tube that was filled with 40 μL lysate buffer and 10 μL protease K. Along with each dissected HCC and DN, surrounding normal liver tissue of the same size was isolated and analyzed as a control. The microcentrifuge tubes were placed in a waterbath (48 °C) to digest the tissue specimens, and then extract DNA using QiaGen kit (Germany). Finally, genomic DNA was confirmed by gel electrophoresis (20 g/L agarose) and stored at −20 °C until using.

### Affymetrix SNP6.0 arrays analysis

The Genome-Wide Human SNP Array 6.0 contains more than 906,600 single nucleotide polymorphisms (SNPs) and more than 946,000 probes for the detection of copy number variation. Each array interrogates SNPs residing on Nsp I or Sty I PCR amplicons that range in size from 200 to 1000 bp. Genomic DNA of 10 fresh matched HCC was send to Shanghai Jingtai Gene Tech Biotechnology Company Limited for SNP6.0 arrays analysis, and performed according to the manufacturer’s commercial protocol (Affymetrix; http://www.affymetrix.com). For PCR, 5 μL of diluted, adapter-ligated DNA and 3.5 μM primer were used in a total volume of 100 μL, and three reactions were prepared for each DNA sample per enzyme. Sixty micrograms of purified product were fragmented and end-labeled using 0.57 mM DLR (GeneChip DNA Labeling Reagent) and 105U of TdT (Promega) for 2 h at 37 °C. Hybridization onto the 250 K Nsp and 250 K Sty EA arrays and subsequent washing steps were done exactly as described by the manufacturer (Affymetrix).

### LOH analysis

128 cases of HCC and 43 DN were analyzed for LOH, along with the surrounding normal liver tissues, by PCR amplification of polymorphic microsatellite markers. 28 microsatellite markers located in 4q, 8p and 16q were selected from the Genome Database (Additional file 1). The reaction mixture was 50μL in volume, containing 1 μL DNA sample, 4 μL of 10 mM dNTP (Gibco BRL, Life Technologies, Inc., Gaithersburg, MD, USA), primers A and B (20 μM each), and 5 μL of 10× buffer, and 2.5U of Taq DNA polymerase (Gibco BRL). The amplification was conducted using a PT-200 thermocycler (MJ Research, Inc., Watertown, MA, USA) for 35 or 25 cycles (95 °C for 40 s, 60 °C for 50 s, and 72 °C for 1 min).

Images of PCR gels were recorded and the intensities of the PCR bands for both alleles were quantified using an image-analyzing system (LabWorks 3.0, UVP, Cambridge, UK). A reduction in fluorescence intensity of 50 % or greater in 1 or more alleles in the HCC and DN (T) compared with the identical allele in the normal liver tissues (N), was defined as an indicator of a LOH.

### Immunohistochemistry

Sections (4-μm) from one representative block for HCC, DN and the corresponding normal tissue from each case were deparaffinized, rehydrated in graded alcohols, incubated with H_2_O_2_ to block the activity of endogenous peroxidase, and then subjected to heat-induced epitope retrieval in 0.1 mol/L citrate buffer at PH 6.0 in a microwave for 20 min. The slides were finally incubated with a primary monoclonal antibody specific for CUB and SUSHI multiple domains-1 (CSMD1) (dilution, 1:200, Boaosen Ltd Company, Beijing, China) for 2 h at room temperature. After incubation with rabbit anti-mouse secondary antibody, a subsequent reaction was performed with biotin-free horse-radish peroxidase enzyme-labeled polymer and visualized using the EnVision plus detection system. The chromogen 3,3′-diaminobenzidine (Dako) was used and sections were counterstained with hematoxylin. Placental tissues were used as positive controls. Nonspecific IgG was used as a negative control. The CSMD1 staining was considered positive when the granular brown reaction was seen in the cytoplasm.

### Cell lines and culture

The human hepatoma cell lines, HepG2 and SK-hep-1, were obtained from Cell Resource Center of Chineses Academy of Medical Sciences and MHCC-97H was provided by Dr. Dang Zheng, the fourth military medical university. The normal human liver cell, HL-7702 was purchased from JENNIO Biological Technology (Guangzhou, China). Cell lines were grown in Dulbecco’s modified Eagle’s medium (Gibco, California, America) supplemented with 10 % fetal bovine serum (Gibco, California, America),100 IU/ml penicillin, and 100 μg/ml streptomycin, at 37 °C in 5 % CO2 atmosphere.

### Quantitative RT-PCR

Total RNA was isolated from HL-7702, HepG2, SK-hep-1 and MHCC-97H cells using the RNA sample Total RNA Kit (TIANGEN, Beijing, China). Aliquots of 1 ug total RNA were reverse transcribed into cDNA using the miScript II RT Kit (QiaGen, Hilden, Germany). Quantitative PCR was run on an ABI 7500 fast Real-Time PCR system using miScript SYBR Green PCR Kit (QiaGen, Hilden, Germany). The reaction cycling conditions were 95 °C for 15 min; 40 cycles at 94 °C for 15 s, 60 °C for 30 s, and 70 °C for 35 s. The CSMD1 Primers used were: 5′-TCCAGTCATTACCACGGCAC-3′ (forward) and 5′-CATGCCCAGCATAGCCATTC-3′ (reverse). The GAPDH primers, an internal normalization control, used were: 5′-GCACCGTCAAGGCTGAGAAC-3′ (forward) and 5′-TGGTGAAGACGCCAGTGGA-3′ (reverse). PCR products were analyzed by electrophoresis on a 8 % acrylamide gel and photographed using Garestream Health GL-2200 PRO.

### Statistical analysis

Statistical analysis was performed using the 2-tailed Fisher exact test or the *χ*^2^ test with the Yates continuity correction. A *P* value of <0.05 was considered statistically significant.

## Results

### The homozygous deletion using SNP6.0 arrays analysis

**Fig. 1:**
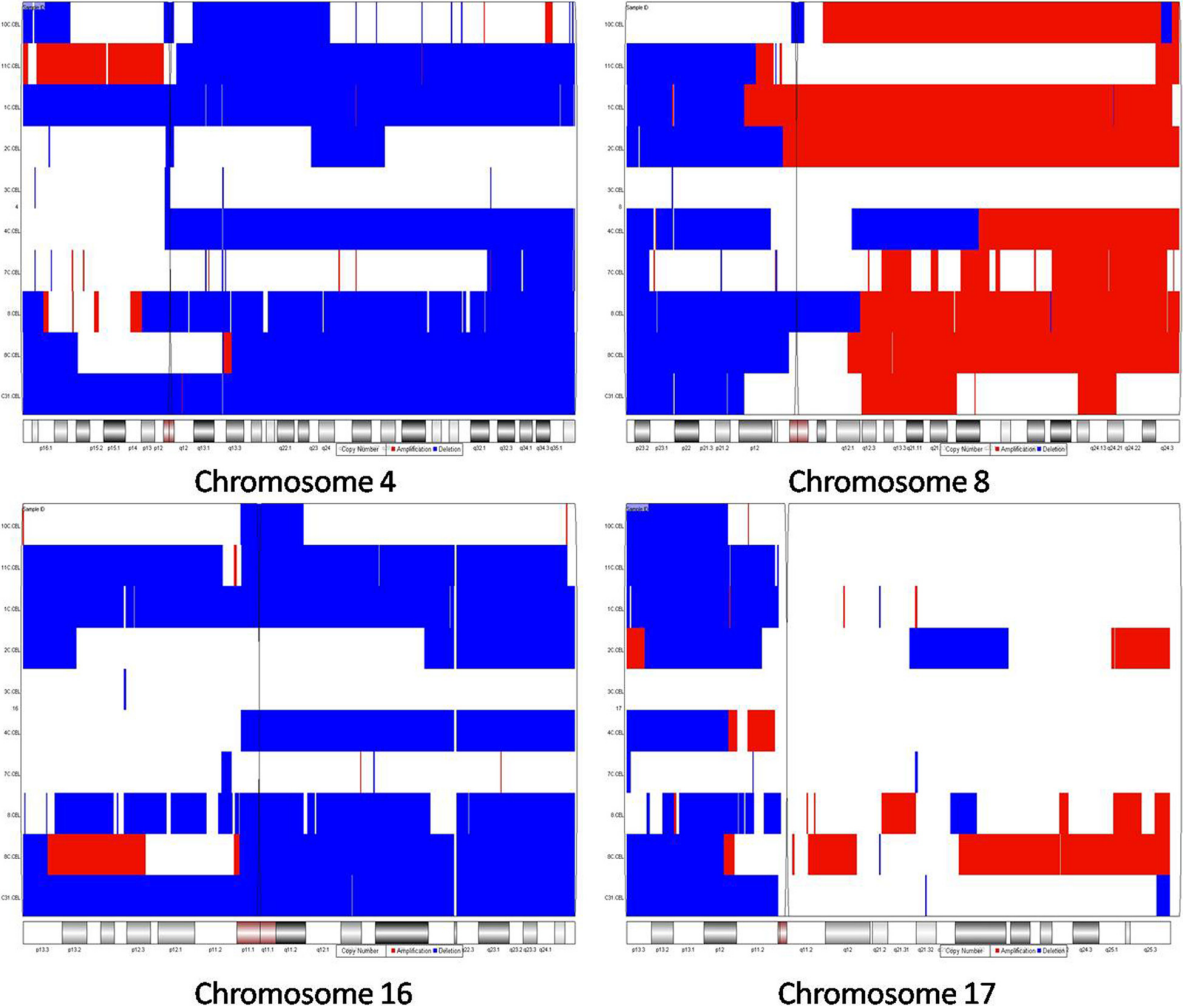
Affymetrix SNP6.0 arrays showed that more than 70 % (7/10) cases had chromosomal deletion on 8p23.2-21.2, 4q13.3-35.1, 17p13.3-12, and 16q11.2- 24.3, respectively. Red color indicated amplification of chromosomal fragment; Blue color indicated neutral LOH without CNV

Affytremix SNP6.0 arrays were applied to 10 matched HCC and the surrounding non-cancerous liver tissues. The results showed some changes for LOH and copy number variation (CNV) in every chromosome. The red color indicated chromosomal amplication, and the blue color represented copy-neutral LOH without CNV. Thus, we found more than 70 % (7/10) cases had chromosomal deletion on 8p23.2-21.2, 4q13.3-35.1, 17p13.3-12, and 16q11.2- 24.3, respectively (Fig. 1). The genes located in these chromosomal fragments included CSMD1, CDH13, NRG1, PCM1, DLC-1, CMIP, and WWOX et al. Because previous studies of LOH have reported that allelic loss of 4q, 8p and 16q are the most frequent chromosomal alteration in a variety of human cancers, including HCC. Thus, we firstly selected the above genes located in the short arm of chromosome 4 and 16, and the long arm of chromosome 8 to investigate further their LOH in turn by a great deal of samples by a PCR-based analysis.

### LOH for HCC

**Fig. 2:**
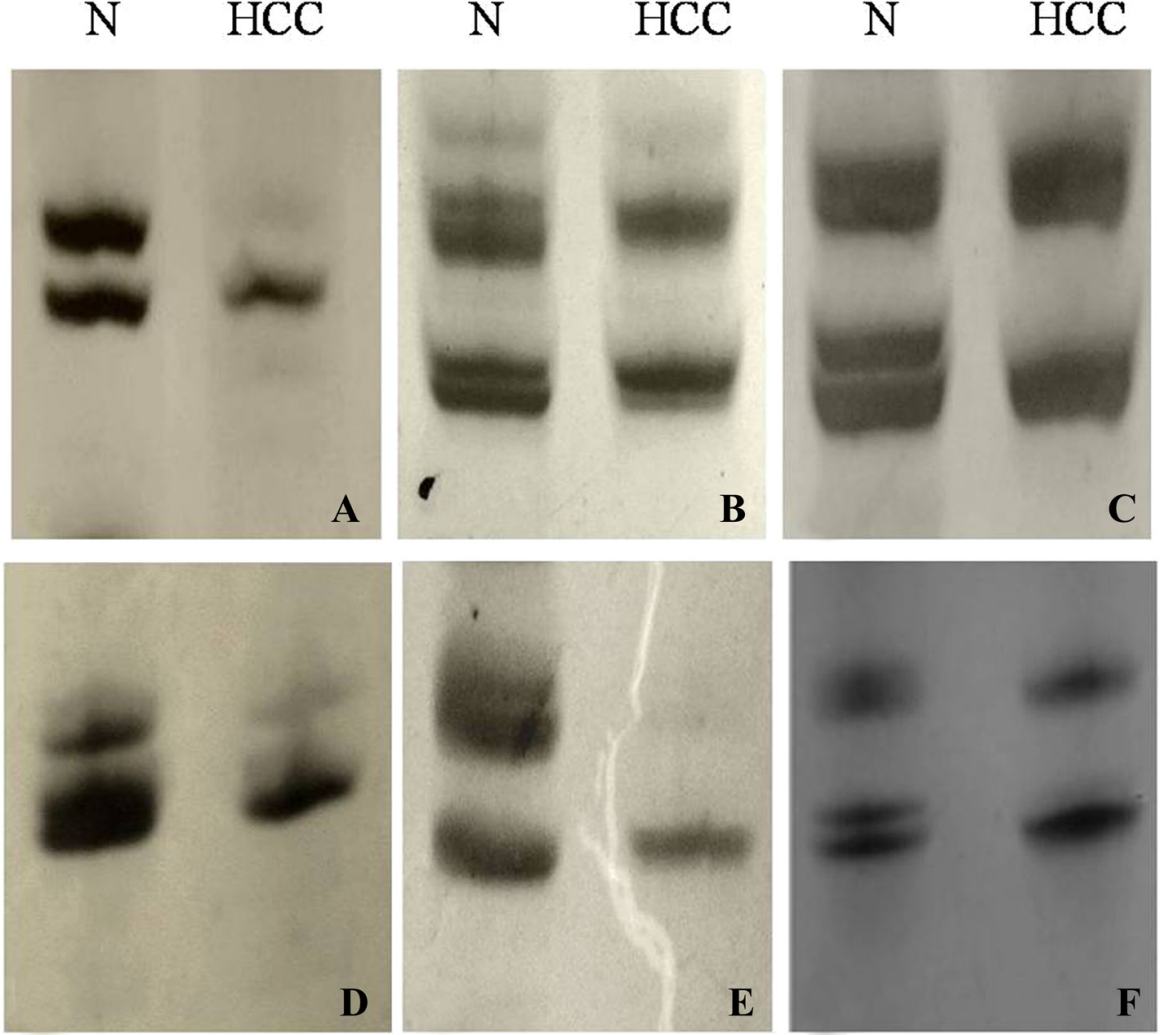
Representative images of PCR gels from a part of microsatellite loci for HCC. Compared with the surrounding normal liver tissues, HCC showed a reduction in fluorescence intensity of 50% or greater in 1 or more alleles. **a**, D4S415; **b**, D4S2954; **c**, D8S262; **d**, D4S3331; **e**, D8S1725; **f**, D8S261. *N* normal liver tissue; *HCC* hepatocellular carcinoma

To date, we examined chromosomal LOH frequency of 128 HCCs at 28 microsatellite markers located in some gene spanning chromosomal band 4q, 8p and 16q. The results showed that LOH frequency (72.7 %, 93/128) at D8S262 for 128 cases of HCC was the highest (Table 1), and the frequency was close to that of SNP6.0 assay. On polyacrylamide gel electrophoresis, there was a reduction in fluorescence intensity of 50 % or greater in 1 or more alleles in the HCC compared with the identical allele in the normal liver tissues (Fig. 2). Moreover, LOH frequency at D8S262 was not associated with sex, age, HCC differentiation, number of HCC, serum HBsAg positivity, or tumor size (Table 2).

**Table 1:** The relationship between the LOH frequency at D8S262 and clinical features of patients with hepatocellular carcinoma

Wk13374861variables	Informative cases (*n*)	LOH (%)	*χ* ^2^	*P*
Age (years)				
≤ 52 (*n* = 68)	49	72.06	2.664	0.103
> 52 (*n* = 60)	44	73.33		
Sex				
Male (*n* = 108)	79	73.15	0.084	0.772
Female (*n* = 20)	14	70		
AFP				
≥ 20 ng/mL (*n* = 76)	58	76.32	1.261	0.261
< 20 ng/mL (*n* = 52)	35	67.31		
HBsAg or HCV				
+ (*n* = 86)	62	72.09	0.042	0.838
- (*n* = 42)	31	73.81		
HCC differentiation				
Grade I (*n* = 14)	9	64.29	0.600	0.741
Grade II (*n* = 78)	57	73.08		
Grade III (*n* = 36)	27	75		
Tumor size				
≥ 5 cm (*n* = 68)	49	72.06	0.026	0.872
< 5 cm (*n* = 60)	44	73.33		
Extrahepatic metastasis				
Presence (*n* = 42)	31	73.81	0.978	0.323
Absence (*n* = 86)	62	72.09

**Table 2:** Relationship between CSMD1 expression and clinical features of patients with hepatocellular carcinoma

Wk13374861variables	CSMD1-positive	CSMD1-negative	*χ* ^2^	*P*
Age (years)				
≤ 52 (*n* = 68)	19	49	0.026	0.872
> 52 (*n* = 60)	16	44		
Sex				
Male (*n* = 108)	29	79	0.084	0.772
Female (*n* = 20)	6	14		
AFP				
≥ 20 ng/mL (*n* = 76)	21	55	0.008	0.930
< 20 ng/mL (*n* = 52)	14	38		
HBsAg or HCV				
+ (*n* = 86)	24	62	0.042	0.838
- (*n* = 42)	11	31		
HCC differentiation				
Grade I (*n* = 14)	6	8	2.856	0.240
Grade II (*n* = 78)	22	56		
Grade III (*n* = 36)	7	29		
Tumor size				
≥ 5 cm (*n* = 68)	20	48	0.312	0.576
< 5 cm (*n* = 60)	15	45		
Extrahepatic metastasis				
Presence (*n* = 42)	11	31	0.042	0.838
Absence (*n* = 86)	24	62		

### LOH for DN

**Fig. 3:**
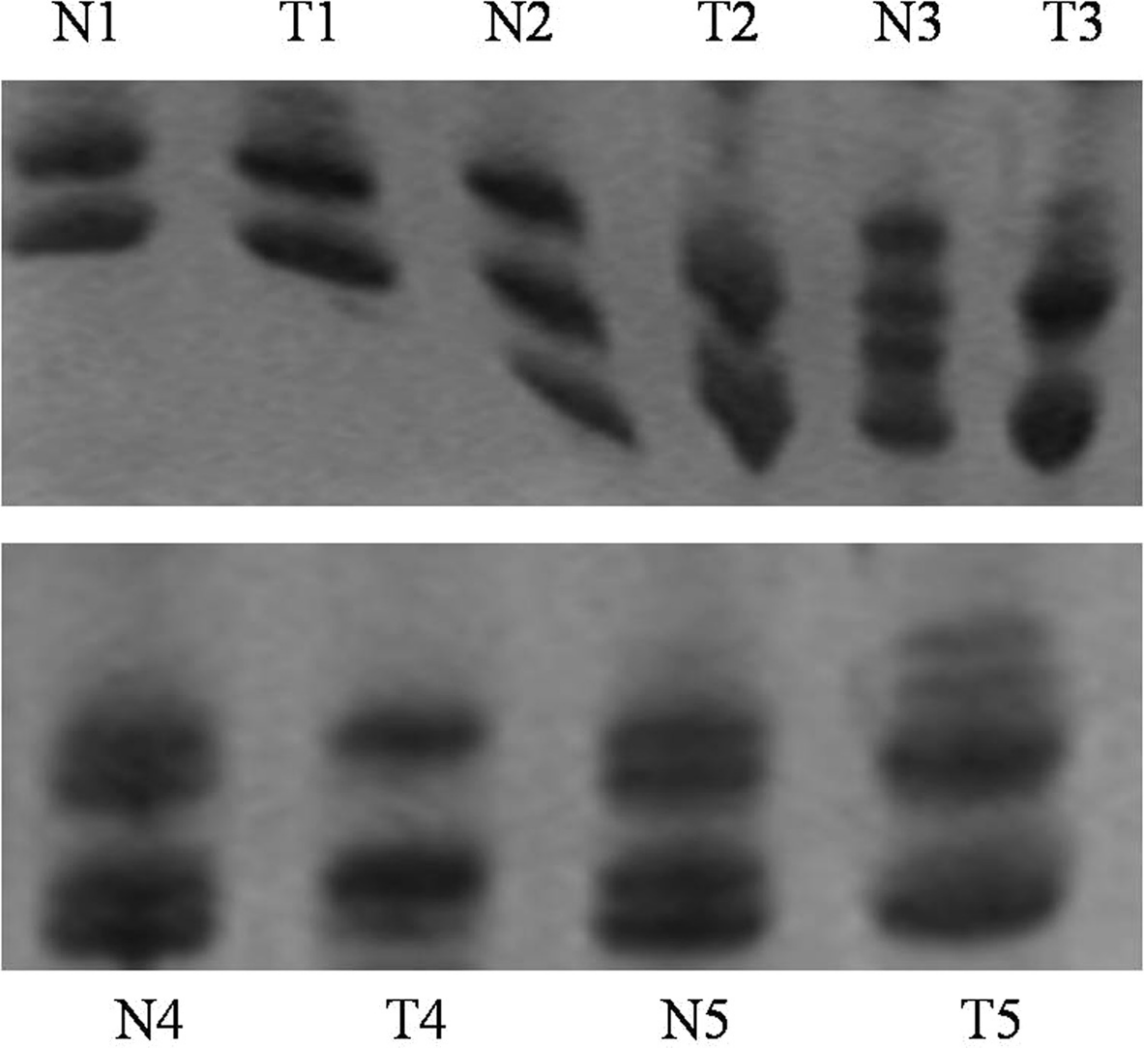
Representative images of PCR gels from a part of microsatellite loci for NAH. Compared with the surrounding normal liver tissues, NAH showed a reduction in fluorescence intensity of 50 % or greater in 1 or more alleles. *N* normal liver tissue; *T* NAH. Among them, T1 had not occurred LOH

Simultaneously, we analyzed chromosomal LOH frequency of 43 DN at the above 28 microsatellite markers. The result showed that the frequency of chromosomal LOH were the highest (51.2 %) at D8S262. Namely, there was also a reduction in fluorescence intensity of 50 % or greater in 1 or more alleles in the DN compared with the identical allele in the normal liver tissues (Fig. 3). These data indicated that chromosomal LOH had occurred in precancerous lesions of HCC.

### The relationship between HCC and its adjacent DN for LOH at D8S262

In the same patient, we found that LOH in HCC was in accordance with its adjacent DN. In other words, LOH was detected in HCC if it was found in its adjacent DN. On the contrary, LOH was not detected in the DN adjacent to HCC if it was found in HCC. Thus, we concluded that D8S262 played an important role in hepatocarcinogenesis.

### CSMD1 expression in normal hepatic tissues, DN and HCC

**Fig. 4:**
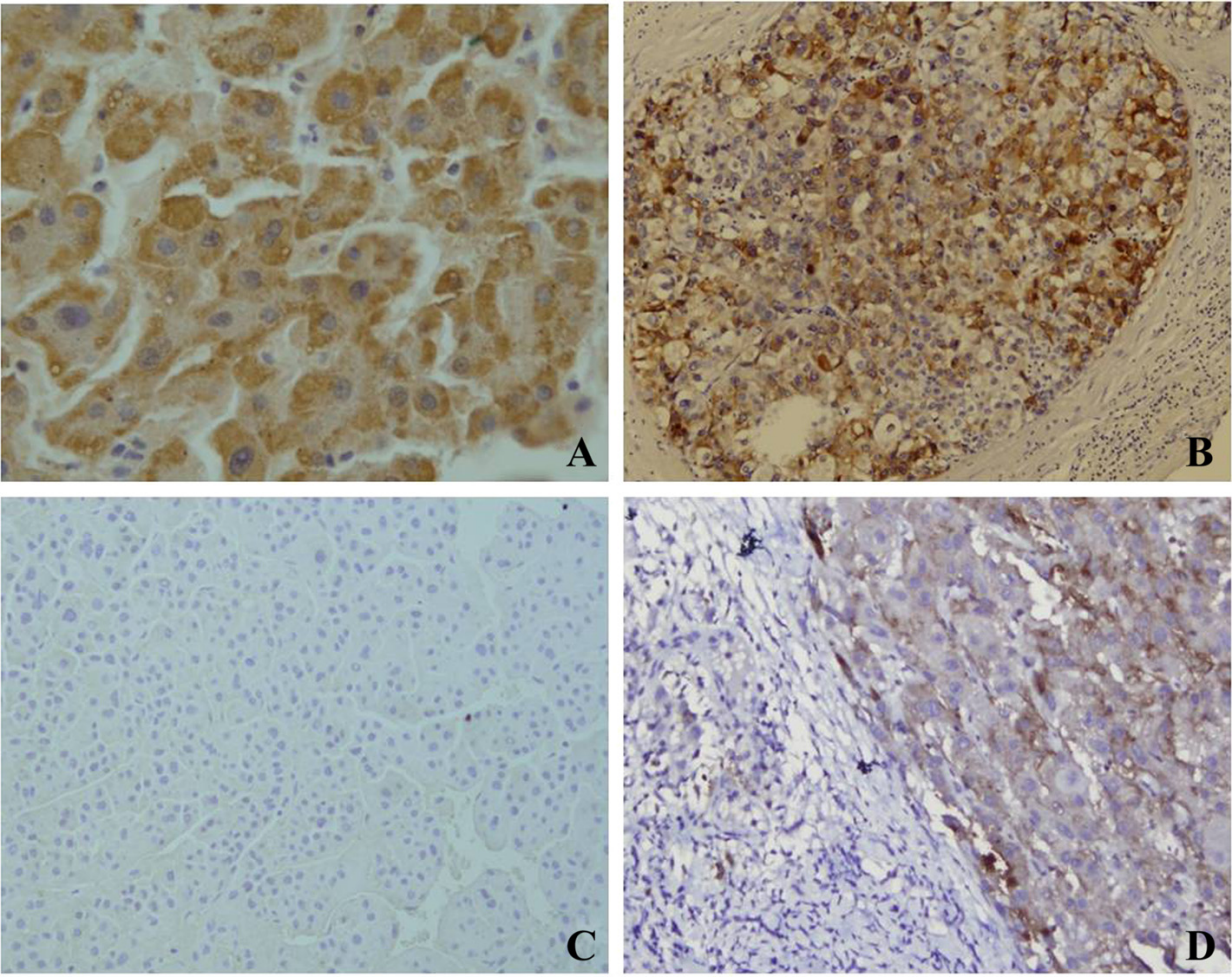
The expression of CSMD1 in HCC, NAH, and the surrounding normal liver tissues. **a**, strong positive expression in normal liver tissue (200×); **b**, positive expression in HCC (100×); **c**, negative expression in HCC (100×); **d**, positive expression in NAH (100×)

**Fig. 5:**
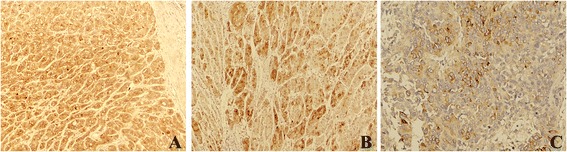
The expression of CSMD1 was stronger in the well-differentiated HCC (**a**, 200×) than that in the moderate (**b**, 200×) and poor differentiated HCC (**c**, 200×)

The staining pattern of CSMD1 was mainly cytoplasmic in 128 cases of HCC and the surrounding normal tissues (Fig. 4), and the positive rate was 27.3 % (35/128) and 82 % (105/128), respectively. The difference reached a statistic significance (*P* < 0.05). The above results demonstrated that CSMD1 might be a TSG for HCC. Moreover, we found the level of CSMD1 expression was higher in the well-differentiated HCC than that in the moderate and poor differentiated HCC (Fig. 5). However, it was not significant in statistics (*P* = 0.240). In 43 DN, 33 (33/44, 75 %) were positive for CSMD1. The positive rate was higher than that (27.3 %) in HCC, but lower than that (82 %) in the surrounding normal tissues.

### CSMD1 expression in HCC cell lines

**Fig. 6:**
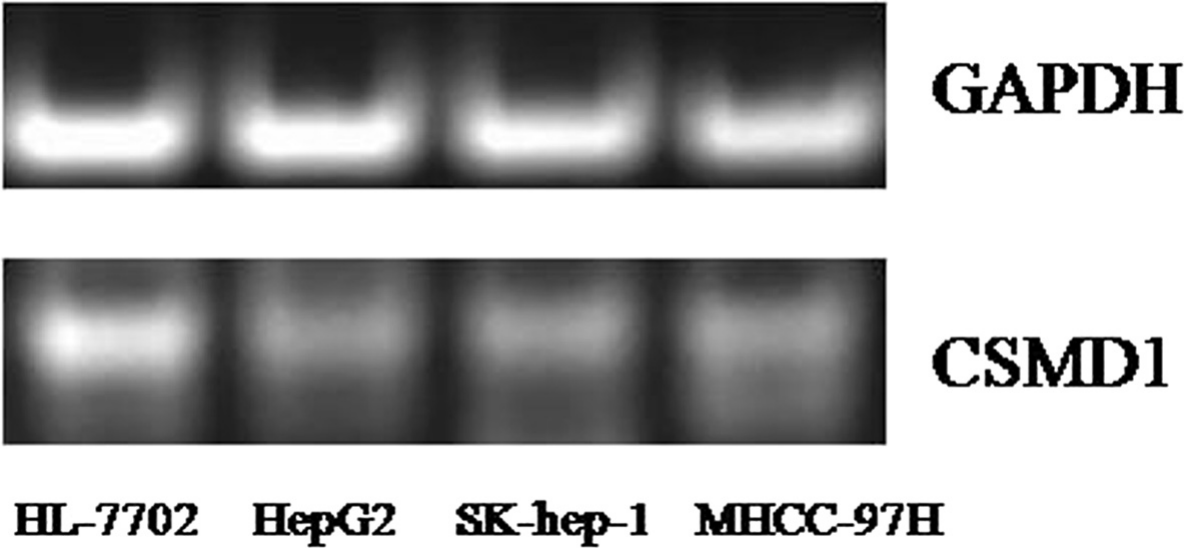
The results of RT-PCR showed that the expression levels of CSMD1 mRNA were down-regulated in HepG2, SK-hep-1 and MHCC-97H compared with that in HL-7702

The results of RT-PCR showed that the expression levels of CSMD1 mRNA were down-regulated in HepG2, SK-hep-1 and MHCC-97H compared with that in HL-7702 (Fig. 6), indicated that CSMD1 played a role of tumor suppression gene.

## Discussion

Similar to other cancers, the carcinogenesis of hepatocellular carcinoma (HCC) is a multi-factor, multi-step, multi-genes and complicated process resulting from the accumulation of sequential genetic and epigenetic alterations. Epidemicly, the main causes of HCC are hepatitis B and C virus infection, dietary exposure to aflatoxin B1 and high-level alcohol consumption. Prolonged exposure to these risk factors is thought to cause an accumulation of chromosomal aberrations and altered gene expression, and eventually results in hepatocarcinogenesis [17–19]. In China, more than 80 % of HCCs develop in patients with chronic infections with HBV. Histopathologically, precancerous lesions of HCC, such as DF, including SCC, DN, and NAH described by Su et al. [3] are often found in cirrhotic liver tissue, and develop into early HCC, which corresponds to *in situ* or microinvasive carcinoma, then develop into progressive HCC through the stage of “nodule-in-nodule”-type HCC. Geneticly, it is considered that LOH regions found in a significant portion of tumors are thought to embody tumor suppressor genes (TSGs). The delineation of such genetic alterations that occur in precancerous lesions and/or early hepatocellular carcinoma (HCC) may be important for monitoring and preventing the occurrence of HCC. However, only few of LOH assays have been reported about precancerous lesions and early HCC [15, 16, 20]. Our previous studies demonstrated that part of DN without SCC and all the DN with SCC in liver cirrhosis tissue were monoclonal hyperplasia, and neoplastic lesion. Moreover, we revealed that there were some changes in DNA copy number in four chromosomal regions in one DN with SCC applying array-CGH [9]. These above results showed that some genetic alteration have occurred in some precancerous lesions of HCC.

Some studies have reported that allelic loss of 4q, 8p and 16q are the most frequent chromosomal alteration in various human cancers. In particular, the loss of 8p23.1-22 is an important event in the initiation or promotion of HCC [21, 22]. We found that there was high frequent loss of chromosomal 4q, 8p and 16q according to the result of Affymetrix SNP6.0 assay. Thus, we selected 28 microsatellite markers at some genes spanning chromosomal band 4q, 8p and 16q to further elucidate the precise location of putative TSGs that might potentially be involved in the tumorigenesis of HCC. The results showed that LOH frequencies at D8S262 for HCC were found to be 72.7 %, which indicated the gene neighboring to D8S262 might be a putative TSG related to the hepatocarcinogenesis. Moreover, many evidences have confirmed this point in many cancers at various levels, including DNA and RNA level [23]. In addition, some studies have demonstrated that LOH is detected on chromosome 8p21.3-p22 in DNs and HCC, and the frequency of LOH is 40.9 % and 42.1 %, respectively [19]. These results suggested that at least one putative tumor suppressor gene involved in the development of HCC might be located on 8p21.3-p22. Therefore, this gene might be related to an early genetic event of hepatocarcinogenesis [20]. We examined LOH frequency at D8S262 for DN in order to investigate further the relationship between DN and HCC. The results demonstrated that LOH frequency at this locus for DN was 51.2 %, which was higher than 40.9 %. This indicated that the event of LOH had occurred in precancerous lesions of HCC, and the gene neighboring to D8S262 might involved in the occurrence and progression of HCC. Simultaneously, it confirmed that DN was a hepatic precancerous lesion, which was coincided in our previous conclusions [9]. The high incidence of LOH observed at an early stage of tumor development was speculated that candidate TSGs located in this region may play an important role in early HCC. CSMD1 located at the neighbor of D8S262. It encodes multiple mRNA transcripts with the largest being 14.3 Kb long. The gene spans over 2 Mb of genomic DNA and contains 71 exons, which encode a 3565 amino acid protein consisting of 14 CUB domains and 28 SUSHI domains. It is a candidate tumor suppressor gene that maps to chromosome 8p23, a region deleted in many tumor types, and has homologies to proteins implicated in carcinogenesis. Moreover, many studies have indicated CSMD1 could be a tumor suppressor gene [23–29]. Thus, according to the above results, we concluded that CSMD1 might be a TSG, and observed the expression of CSMD1 in HCC, DN and the surrounding liver tissues by immunohistochemical staining methods. The results showed that the positive rate of CSMD1 increased in turns in them. This indicated that CSMD1 might be a TSG related to the occurrence of early HCC. In addition, RT-PCR results demonstrated that the expression levels of CSMD1 were down-regulated in HCC cell lines compared with human normal hypertocyte cells HL-7702. Of course, it will need to be confirmed by a great deal of studies. Interestingly, Mohamed et al. [23] studied the expression pattern of the CSMD1 protein in invasive ductal carcinoma (IDC), and found that reduction of CSMD1 expression was significantly associated with high tumor grade. Their results supported the idea that CSMD1 was a tumor suppressor gene. Midorikawa et al. [28] found that homozygous deletions frequently on 8p23.2, and mRNA expression of the extremely large gene CSMD1 within this region was decreased in overt HCC, suggesting that CSMD1 plays a pivotal role in liver cancer progression. Our results showed that the expression of CSMD1 in the surrounding normal liver tissues was obviously higher than that in HCC, and reached a statistic significance. Moreover, the level of CSMD1 expression was higher in the well-differentiated HCC than that in the moderate and poor differentiated HCC though there was not a significant difference. Thus, we concluded that CSMD1 might a TSG according to our results. Lu et al. [21, 22] also found that the LOH at 8p23.2-21 was significantly more frequent in the metastatic than corresponding primary tumor lesions in individual HCC cases, and the significant difference between them suggests that deletion of 8p23.2-22 may be not only an early event in the initiation of HCC but also involved in subsequent tumor aggressiveness, especially in the metastatic progression of HCC. Moreover, they considered that some unknown genes located adjacent to the markers D8S262, D8S1819 to D8S1109 and D8S261 play an important role in HCC. According to the above results, we found the conclusions were different for the role of CSMD1 in HCC. This may be related to the difference of people population. However, we also concluded at least one critical TSG lie at the restricted minimal regions at 8p23.2-22, and notably, CSMD1 may be one of them. At present, we considered it was a TSG related to early HCC. Of course, we need identify the gene by more studies.

## Conclusions

Our results demonstrated that there were similar genetic changes between DN and HCC. Moreover, LOH frequencies at D8S262 for DN and HCC were the highest, and the expression of CSMD1 was obviously lower in HCC than that in the surrounding liver tissue. Thus, we considered that CSMD1 may be a critical TSG associated with an early genetic event of hepatocarcinogenesis, and a predictor for the monitor and prevention of HCC. Of course, more studies need to be performed.

## Additional file

Additional file 1:
**The information of 28 microsatellite markers located in 4q, 8p and 16q selected from the Genome Database.**

## Abbreviations

DF: dysplastic foci
DN: dysplastic nodules
FAH: Foci of altered hepatocyte
GSF: glycogen-storing foci
HCC: hepatocellular carcinoma
LOH: loss of heterozygosity
SCC: small cell change
LCC: large cell change
LGDN: low grade dysplastic nodules
HGDN: high grade dysplastic nodules
MCN: mixed cell nodules
NAH: nodules of altered hepatocyte
SNP: single-nucleotide polymorphism
TSG: tumor suppressor genes
