# Supplementary material for: Loss of heterozygosity at D8S262: an early genetic event of hepatocarcinogenesis
Source: Diagn Pathol. 2015 Jun 16;10:70. doi: 10.1186/s13000-015-0308-y (PMC4469120; doi:10.1186/s13000-015-0308-y)
Supplement: Additional file 1: — The information of 28 microsatellite markers located in 4q, 8p and 16q selected from the Genome Database. [file 13000_2015_308_MOESM1_ESM.doc]

Additional file 1. The information of 28 microsatellite markers located in 4q, 8p and 16q selected from the Genome Database

| Microsatllite marker | Forward primer | Reverse primer |
| --- | --- | --- |
| D8S262 | **AGCTCAAAAGCGAAGGTGAT** | **GGCAACAAAGTGAGATCCTG** |
| D8S1788 | **AGTTCAAGCCTAGTTTCATAAAAG** | **AAGACTCCTAATGTCTATTTTTCAG** |
| D8S1781 | **CACTGGGGGCTAACCTT** | **TTTTGAATCGTGTCAGCA** |
| D8S1806 | **GACTGGAAGATAACATTTTCAAAC** | **CCTAACTCTGTGACTCTGTGTGT** |
| D16S518 | **GGCCTTTTGGCAGTCA** | **ACCTTGGCCTCCCACC** |
| D16S3049 | **GCAATGAAGGCAACAAAGT** | **TTAAAAGACCTGGGGGAAT** |
| D16S3096 | **GATCTGGCTTACGATGATTTCTAAC** | **CCGTGATGATGTCTGCAAC** |
| D16S3029 | **ATAGAGTTGGGCTGCATAGA** | **CTTTCCTGAAATTGGAAGTGA** |
| D16S504 | **AGCTTGTTCAGGGAAACC** | **CAGGGATGTAGGACGTAGG** |
| D16S684 | **GGCCAAAAAAGCAGATTGG** | **TGGAATTTGAGTGGCTTTCT** |
| D16S501 | **CTGNCCCTACTCTGCCACAC** | **GGAACCTGCCCATGGAGTGT** |
| D16S402 | **TTTTGTAACCATGTACCCCC** | **ATTTATAGGGCCATGACCAG** |
| D8S261 | **TGCCACTGTCTTGAAAATCC** | **TATGGCCCAGCAATGTGTAT** |
| D8S499 | **GGGTGACAGAGCGAGATTC** | **CCTTGCCATTTTCACACGTG** |
| D8S1125 | **CCCCCTAAAATTTAGCTCCA** | **TATGCCTAGCCCTCCTTTCT** |
| D8S1810 | **ATGATGCTGAGTCCCCA** | **CAGAGGGCTGATTTTATGC** |
| D8S1827 | **GACAGAATCATGTGGCCTTT** | **TTTTGTAAAATGTAAAATTGGCTTT** |
| D16S422 | **CAGTGTAACCTGGGGGC** | **CTTTCGATTAGTTTAGCAGAATGAG** |
| D16S511 | **CCCCGGAGCAAGTTCA** | **CAGCCCAAAGCCAGATTA** |
| D4S415 | **GGGCTAAGGCAACTCC** | **GGTTAGATTAACTGCAAAACG** |
| D4S3331 | **TGTAGATAGATAGGTAGGTAGCAGG** | **GTTTACCCTTATATCAGCTAACCC** |
| D4S3030 | **AGCTCCTAATAAAGAATAGCCG** | **ACTCAAGCAGAACTGATGATG** |
| D4S2954 | **CCATTTCAGTGTCTGTGACTA** | **GGAAGCCAATTCCTCATA** |
| D8S1810 | **ATGATGCTGAGTCCCCA** | **CAGAGGGCTGATTTTATGC** |
| D8S1725 | **ACACCTACCAAGGACTGCTG** | **CGGGCTGGGACAATTTT** |
| D8S552 | **AGGATTGTAATTTCCTTGC** | **GGGACTTTTTGAAGGTTTG** |
| D8S1827 | **GACAGAATCATGTGGCCTTT** | **TTTTGTAAAATGTAAAATTGGCTTT** |
| D8S1754 | **CAGGGAAGTCTCGGTTTG** | **TCAGGGACACGATTCAGC** |
